# Individual and Situational Predictors of Threatening Dream Content During the COVID‐19 Pandemic

**DOI:** 10.1111/jsr.70336

**Published:** 2026-04-02

**Authors:** Ville Loukola, Jarno Tuominen, Eveliina Malinen, Katariina Tienhaara, Henri Olkoniemi, Antti Revonsuo, Katja Valli

**Affiliations:** ^1^ Department of Psychology and Speech‐Language Pathology, and the Turku Brain and Mind Center University of Turku Turku Finland; ^2^ Unit of Psychology University of Oulu Oulu Finland; ^3^ Department of Cognitive Neuroscience and Philosophy University of Skövde Skövde Sweden; ^4^ Department of Medicine, Sleep and Breathing Center Turku University Hospital Turku Finland

**Keywords:** consciousness, dreaming, subjective experiences, threat simulation theory

## Abstract

Previous studies have examined how the COVID‐19 pandemic affected dream recall, dream content, and nightmares. However, relatively little attention has been devoted to the individual and situational factors associated with pandemic‐induced changes in dreams. The threat simulation theory of dreaming predicts that threatening situations in our waking life (situational factors) influence threatening dream content. At the same time, individual differences predispose some people to be more prone to experiencing threatening dreams more frequently than others. Using a large Finnish sample of prospective dream diaries, we analysed the relative importance of individual (e.g., belonging to a COVID‐19 risk group, life satisfaction, depression and anxiety symptoms) and situational factors (e.g., daily COVID‐19 worry, COVID‐19 media consumption, negative and positive emotions) to determine the best predictors for threatening events and COVID‐19‐related threatening events in dreams. Random forest analyses revealed that individual factors were consistently better predictors than situational factors for both threatening events and pandemic‐related threatening events in dreams. Lower life satisfaction was the only statistically significant predictor of threatening events and experiencing fewer positive emotions in the past 2 weeks was the only statistically significant predictor of pandemic‐related threatening events in dreams. These findings suggest that the propensity to experience threatening dream content, including pandemic‐related threatening events, is more of a stable trait rather than a daily fluctuating feature of dreams. In light of the threat simulation theory, it could be argued that individual variation in the proneness to simulate threatening events adaptively interacts with daily experiences to modulate threatening dream content.

## 1 Introduction

The COVID‐19 pandemic had a large impact on the mental and physical well‐being of individuals across the world. It not only affected people's daily lives but also their dreams, as addressed by numerous studies that have investigated how the COVID‐19 pandemic impacted dream recall, dream content, and nightmares (for review, see Gorgoni et al. 2022). In general, the findings on whether the COVID‐19 pandemic increased negatively toned dreams and nightmares are mixed, with retrospective questionnaire studies indicating a negative impact on dreaming (e.g., Musse et al. 2020; Scarpelli et al. 2021; Schredl and Bulkeley 2020; Simões et al. 2022), while prospective dream diary studies have been more inconclusive, with several studies noting only a meagre or no effect (Domhoff 2022; Koppehele‐Gossel et al. 2023; Loukola et al. 2024; Scarpelli et al. 2022). Methodological variations, particularly the use of pandemic‐focused questions and the susceptibility of questionnaire studies to memory and reporting biases, may account for these discrepancies. Many questionnaire studies on COVID‐19 explicitly requested people to compare their dreams or nightmares before and during the pandemic, which may have biased the findings towards recent negative dreams with pandemic content. Because negatively toned, bizarre, and salient dreams are generally easier to recall than neutral or mundane ones, retrospective questionnaires tend to underrepresent everyday dreams and overrepresent negative and nightmarish content (Loukola et al. 2024). Moreover, questionnaires may capture people's self‐conception as dreamers and their attitudes towards dreams rather than their actual dream content (Sikka 2020). Whereas questionnaires allow collecting data from substantially larger samples, the more labour‐intensive dream diaries provide a more ecologically valid assessment of daily dream experiences.

However, relatively little attention has been devoted to investigating the individual and situational factors associated with pandemic‐induced changes in dreams. Dreams about the pandemic have been found to be associated with older age (Giovanardi et al. 2021), younger age (Simões et al. 2022; Wang et al. 2020), female gender (Schredl and Bulkeley 2020), belonging to a COVID‐19 risk group (Simões et al. 2022), COVID‐19 related worry (Kennedy et al. 2022; Simões et al. 2022; Wang et al. 2020), mental health issues (Giovanardi et al. 2021; Simões et al. 2022), self‐reported employment problems (Simões et al. 2022), higher education level (Schredl and Bulkeley 2020), worsened sleep and middle‐of‐the‐night insomnia (Kennedy et al. 2022) and death of a significant person (Simões et al. 2022). Musse et al. (2020) concluded that younger age, pre‐existing psychiatric conditions, and use of sleeping medication are associated with nightmares about the pandemic. A key limitation of these studies is that they have utilised questionnaires or the most‐recent‐dream method, which are retrospective in nature and do not provide a representative sample of dream content (Sikka 2020). At this time, prospective dream diary studies that would address the relative importance of individual and situational factors in predicting pandemic‐related threatening dream content are lacking.

According to the *threat simulation theory* (TST), dreams are simulations during sleep where the dreamer rehearses situations that could endanger their well‐being in waking life (Revonsuo 2000). This nocturnal training enhances individuals' threat perception and recognition skills and makes their reactions to the threatening events more effective in similar subsequent waking life situations. This training enhanced the fitness of ancestral humans and, therefore, was selected for. There are indications of this kind of training effect from lucid dreaming to waking‐life (e.g., Erlacher and Schredl 2010) although no studies have examined how responses to threats in dreams would generalise to the waking environment. Of note, the nocturnal simulation mechanism is still evidently active in the current environment (Revonsuo and Valli 2000), although dreams may no longer provide an effective rehearsal function, given the mismatch between the original environment of evolutionary adaptedness and the modern world.

TST predicts that situational factors, such as encountering a threatening event in waking life, affect threatening dream content. Personally experienced threatening events in waking life seem to most powerfully modulate subsequent threatening dream content, most evidently seen in posttraumatic dreams (e.g., Mellman et al. 2001; Valli et al. 2005). However, stress (Lafrenière et al. 2018) and rumination on anxious thoughts before sleep (Feng and Wang 2022) also increase threatening events in dreams. Further, higher amounts of media consumption about a threatening event seem to alter dreams by increasing threatening dream content (Propper et al. 2007). All these observations can be interpreted to offer support for TST.

TST also predicts that there are individual differences in how sensitive or prone persons are to simulate threatening events in their dreams. For the threat simulation mechanism to be selected for, variation in the trait needs to exist in the population. Being prone to simulate a higher number of threats in dreams must have yielded an adaptive advantage in some environments. This could be due to innate characteristics or features of the ontogenetic environment (e.g., high environmental threat pressure from birth). Personality traits are moderately to highly heritable, and neuroticism is strongly associated with nightmare frequency (Schredl and Göritz 2021). Further, hyperarousal and impaired fear extinction caused by traumatic experiences and childhood adversity are suspected to be predictors of nightmare disorder (Gieselmann et al. 2019).

Notably, TST is not the only theory about dream content that makes similar predictions about the effect of the pandemic on dreaming. The continuity hypothesis proposes that dream contents reflect waking life and waking personality traits, but without any evolutionary functions or any specific selection mechanisms for threatening events (Schredl and Hofmann 2003). Emotion regulation theories of dreaming propose that dreaming helps us process and integrate negative or threatening emotional experiences by reactivating and recontextualizing them in a relatively safe, offline simulation during sleep, with nightmares reflecting a failure of this function (e.g., Cartwright 2012; Levin and Nielsen 2009).

Dream content seems to be more impacted by relatively stable trait factors than daily fluctuating state factors, although studies have predominantly focused on nightmare frequency rather than general dream characteristics. Kelly and Mathe (2024) found that after accounting for boundary thinness and nightmare proneness (trait factors), state distress did not predict nightmare frequency. Gratton et al. (2024) observed that trait anxiety predicted higher nightmare frequency. Moreover, Samson‐Daoust et al. (2019) reported that higher trait anxiety predicted a more negative emotional tone of dreams (but daily stress did not). Sikka et al. (2023) found, in a large sample of dreams collected during the COVID‐19 pandemic, that higher daily COVID‐19 worry was associated with more negative affect in dreams in between‐person level but not in within‐person level. Importantly, trait and state factors likely interact in modulating dream content, and trait factors influence how strongly one is affected by state factors (Levin and Nielsen 2009). Individual stable trait factors set a baseline pattern that determines how our dream content is modified by situational fluctuating factors, such as our daily experiences, mood, or stress levels. In support of this, Blagrove and Fisher (2009) noted that nightmare frequency is associated with trait factors as a function of state (daytime mood) but only in participants who scored high on thin psychological boundaries. However, one study indicates a totally opposite effect, that is, state stress moderates the effect of trait neuroticism on nightmare frequency (Schredl 2003), although these results were not replicated in a later sample (Gessert and Schredl 2023).

### 1.1 The Aims and Hypotheses of the Present Study

In this study, we examined, in a large sample of Finnish prospective dream diaries, how multiple individual and situational factors predict threatening events and pandemic‐related threatening events in dreams. Preregistration for all the hypotheses and statistical methods can be found at the study's Open Science Framework registration page: https://osf.io/jf5yg/.

We opted to use the terms *individual* and *situational* factors instead of the terms *trait* and *state* factors because some individual factors in this study are not traits (e.g., anxiety or depression symptoms in the last 2 weeks), and some situational factors are not mental states (e.g., consumption of COVID‐19‐related media). We utilised TST as the theoretical framework, although it does not differentiate which factors should be the best predictors for threatening dream content in the context of the COVID‐19 pandemic. By testing both individual and situational factors in a large sample of prospective dream diaries, we are able to provide additional insight into how trait versus state factors are predictive of dream content. Additionally, we can expand research on TST as the field is currently lacking a detailed analysis on factors predictive of threatening dream content in general, and pandemic‐related threatening dream content in particular, given that previous research has been done mostly on nightmares (Blagrove and Fisher 2009; Gratton et al. 2024; Kelly and Mathe 2024; Levin and Nielsen 2009; Schredl 2003) or on dream affect (Samson‐Daoust et al. 2019; Sikka et al. 2023), not threatening dream content.

We expected that a greater number of threatening events (H1) and pandemic‐related threatening events (H2) in dreams would be associated with the following individual factors: belonging to the COVID‐19 risk group, a close person belonging to the COVID‐19 risk group, lower life satisfaction and peace of mind, a greater amount of negative emotions and a lower amount of positive emotions in the last 2 weeks, and greater anxiety or depression symptoms in the last 2 weeks. We further expected that a greater number of threatening events (H1) and pandemic‐related threatening events (H2) in dreams would be associated with the following situational factors: a greater amount of negative emotions and a lower amount of positive emotions during a mind‐wandering task the previous evening, a greater amount of negative emotions and a lower amount of positive emotions during the day preceding the dream report, a greater amount of COVID‐19 related worry experienced during the previous day, having or fearing to have COVID‐19 or a close person having or fearing to have COVID‐19, and a greater amount of COVID‐19‐related media consumption during the previous day.

## 2 Methods

### 2.1 Participants and Procedure

The ‘Covid‐on‐Mind’ study investigated the effects of the COVID‐19 pandemic on the mind via dreams and mind wandering. The data were collected between the 16th of April and the 11th of June 2020, coinciding with the middle of the first wave of the pandemic and during the time the restrictions in Finland were the strictest. See Loukola et al. (2024) for the detailed procedure of the study. Briefly, the participants were recruited via social media and through an email newsletter that was sent to the student societies of almost all the major universities in Finland. The study also gained mainstream media attention, which enabled recruiting participants from the general population. The data were collected anonymously via Webropol Surveys with personal participant IDs to connect different responses to the same individual. The participants first filled in a general well‐being questionnaire about psychological well‐being and ill‐being, sleep quality, dream experiences, and COVID‐19‐related experiences, and then kept a dream diary and engaged in a daily mind‐wandering task for 2 weeks. In the dream diaries, the participants were asked to report, immediately after awakening, all the dreams they remembered, as truthfully and in as much detail as possible.

A total of 1534 dream reports and 1251 mind‐wandering reports were submitted by 297 participants. In this study, we only used data from participants who were at least 18 years old, provided consent to participate, answered the well‐being survey, and provided at least five dream and mind‐wandering reports. Because daily situational factors were measured in conjunction with the evening mind‐wandering reports, a minimum of five reports was utilised to gain sufficient information on the effect of situational variables on subsequent dreams. No participants were excluded based on, for example, mental health symptoms or poor sleep quality. This brought the total sample size to 85 participants (4 men, 78 women, and 3 others; M = 37.71 years, SD = 15.98, range = 19–73). The participants provided 1129 dream reports, on average 13.29 dreams (SD = 6.86, range = 5–40). Mind‐wandering reports are not analysed in this study.

### 2.2 Materials

#### 2.2.1 Independent Variable: Individual Factors

Individual factors were measured in the well‐being questionnaire at the beginning of the study, prior to the dream report collection. *Belonging to a COVID‐19 risk group* was measured with a binary yes/no question ‘Do you belong to a COVID‐19 risk group: over 70 years old, chronic diseases that severely weaken the capabilities of lungs, heart, or immune system, severe obesity (BMI > 40), daily smoking?’ (15.3% responded ‘yes’, *n* = 13). *A close one belonging to a COVID‐19 risk group* was measured with a binary yes/no question ‘Do your close ones belong to a COVID‐19 risk group: over 70 years old, chronic diseases that severely weaken the capabilities of lungs, heart, or immune system, severe obesity (BMI > 40), daily smoking?’ (72.9% responded ‘yes’, *n* = 62). Life satisfaction was measured with the *Satisfaction with Life Scale* (SWLS; Diener et al. 1985). The scale score varies between 5 and 35, with a higher score indicating greater life satisfaction. The sample mean suggests that the participants were, in general, ‘slightly satisfied’ and very close to the general population (Pavot et al. 1991). Negative and positive emotions in the last 2 weeks were measured with the *Positive and Negative Affect Schedule* (PANAS; Watson et al. 1988) with the instruction ‘Assess to what extent you have experienced the following feelings during the past 2 weeks’. The questionnaire scores for both positive and negative emotions vary between 10 and 50, with a higher score indicating more positive/negative emotions. The sample mean was very close to the general population (Watson et al. 1988). Peace of mind was measured with the *Peace of Mind Scale* (Lee et al. 2013). The scale score can range from 1 to 5, with higher scores indicating higher peace of mind. The sample mean was very close to the average in Asian American and Taiwanese populations (Lee et al. 2013). Anxiety symptoms in the last 2 weeks were measured with the *Generalised Anxiety Disorder* questionnaire (GAD‐7; Williams 2014) with the instruction ‘How often have you had these problems in the previous 2 weeks?’ Questionnaire scores can range from 0 to 21, with higher scores indicating more frequent anxiety symptoms. The sample mean was on the cut‐off point of mild and moderate anxiety, and it indicates slightly higher anxiety compared to a large German general population sample (Hinz et al. 2017). Depressive symptoms in the last 2 weeks were measured with the *Patient Health Questionnaire* (PHQ‐9; Kroenke et al. 2001) with the question ‘How often have you had these problems in the previous 2 weeks?’ Questionnaire scores can range from 0 to 27, with higher scores indicating more frequent depressive symptoms. In this sample, the mean was on the cut‐off point of mild and moderate depression, and it indicates slightly higher depression compared to a large German general population sample (Hinz et al. 2016).

#### 2.2.2 Independent Variable: Situational Factors

Situational factors were measured daily in conjunction with the evening mind‐wandering reports. Because the participants did not always complete the mind‐wandering report in the evening before reporting dreams, the sample sizes for different variables differ from the total sample size of dreams. Additionally, since the participants did not always complete the entire questionnaire, the sample sizes vary for different situational factors. Further, a night may include multiple dream reports, but only one situational factor value from the preceding evening. Thus, some situational factor values correspond to multiple dreams. *Negative and positive emotions during mind wandering* were measured with the question ‘How much did you experience negative/positive emotions during the (mind‐wandering) task’? with a 5‐point Likert scale (1 = ‘not at all’ and 5 = ‘very much’). Negative and positive emotions during the day were measured with the *Positive and Negative Affect Schedule* (PANAS; Watson et al. 1988) with instructions ‘Assess to what extent you have experienced the following feelings during the day.’ The questionnaire scores for both positive and negative emotions vary between 10 and 50, with a higher score indicating more positive/negative emotions. The sample mean was very close to the general population (Watson et al. 1988). COVID‐19‐related worry was measured with a question ‘How much has coronavirus worried you today?’ with a 5‐point Likert scale ranging from ‘not at all’ to ‘very much’. The sample mean indicates low worry. Having or fearing to have COVID‐19 was measured with a question ‘Have you had or do you suspect having COVID‐19?’ with options ‘I have had COVID‐19’ (1.3%, *n* = 13), ‘I'm afraid that I have COVID‐19’ (6.1%, *n* = 59), and ‘I haven't had and I'm not afraid of having COVID‐19’ (92.6%, *n* = 900). A close person having or fear of having COVID‐19 was measured with a question ‘Have your close ones had or do you suspect them having COVID‐19?’ with options ‘Has had COVID‐19’ (6.3%, *n* = 61), ‘I'm afraid that they have COVID‐19’ (5.3%, *n* = 51), and ‘A close person of mine hasn't had or I'm not afraid that has COVID‐19 at the moment’ (88.4%, *n* = 857). COVID‐19‐related media consumption was measured with a question ‘How much have you followed coronavirus‐related news today?’ with options ‘not at all’, ‘under 30 min’, ’30 min to 1 h’, ‘1 to 3 h’, and ‘over 3 h’. The mean indicates that the participants had followed COVID‐19‐related media daily on average between 0.5 and 3 h.

#### 2.2.3 Dependent Variables: Threatening Events and COVID‐19‐Related Threatening Events in Dreams

Threatening events in dreams were measured with the *Dream Threat Scale* (Revonsuo and Valli 2000), a content analysis tool for identifying and categorising threatening events in written reports. A detailed description of how the scale was utilised in this sample is available in Loukola et al. (2024), but briefly, threatening events were identified and categorised by three independent judges with acceptable interrater agreement levels. Additionally, in the pandemic‐related dreams, threatening events that were specifically related to COVID‐19 were extracted by V.L. and K.V. from the previously identified threats, and any ambiguous cases were discussed until agreement was reached. A detailed description of how pandemic‐related dreams were identified is available in Loukola et al. (2024).

### 2.3 Statistical Analyses

Previous literature does not specify which independent variables best predict dream content. Many of our predictors were highly correlated (see Table S1), making it inappropriate to include them all in the same mixed‐effects model due to multicollinearity. Testing each predictor separately would increase the likelihood of Type I error, and we had no theoretical basis to select only certain predictors, especially since TST predicts that both situational and individual variables may influence threatening dream events. To mitigate this, we used the random forest method to objectively assess variable importance (see Kuperman et al. 2018). This non‐parametric technique builds multiple decision trees using random subsets of predictors and observations to estimate which variables best explain the outcome. Random forest plots focus on the most important variables but do not indicate the direction of the effect or statistical significance. Thus, the random forest method was used to choose the fixed effects for the mixed‐effects models for H1 and H2. Predictors with the highest variable importance—just before a sharp drop—were selected as fixed effects. The cut‐off point is indicated by a black line in the random forest plots. To ensure representative data for both individual and situational factors in the random forest analyses, participants who failed to provide at least three consecutive measures related to the mind‐wandering task and the following night's dream report were excluded. This resulted in a final sample of 83 participants (4 men, 76 women, 3 others; M_age_ = 37.65, SD_age_ = 15.85), who contributed 1115 dream reports (M = 13.45 per participant, SD = 6.87). The excluded participants were included in the mixed‐effects models as they can better handle missing data (Baayen et al. 2008).

After selecting the predictors, we built generalised linear mixed‐effects models for H1 and H2 using the *glmmTMB* package (Brooks et al. 2022) in R (Version 4.4.0; R Core Team, 2024), which can effectively handle the zero inflation of dependent variables. Notably, 45.0% of dreams contained no threats, and 94.2% contained no COVID‐19‐related threats, resulting in a substantial number of zero values. *DHARM*a package (Hartig 2024) was used to test zero‐inflation and overdispersion. Categorical variables (i.e., self‐belonging or a close person belonging to the COVID‐19 risk group) were fitted as deviation coded fixed effects. Continuous variables were centred. Because previous research has indicated that younger people and women have more negatively toned dreams (e.g., Scarpelli et al. 2021), age and gender were controlled by fitting them into the models as fixed effects (age was centred and gender deviation coded). We applied a maximal‐that‐converges random effects structure in the models with participants fitted as random intercepts (Barr et al. 2013). Precise *p*‐values cannot be calculated for mixed‐effects models due to difficulty in determining exact degrees of freedom (Baayen et al. 2008). Thus, statistical significance at the 0.05 level is indicated by values of *z* > |1.96|. Complete models are reported in the Tables S2 and S3.

## 3 Results

### 3.1 Descriptive Statistics for the Individual and Situational Factors

Descriptive statistics for the individual factors are shown in Table 1, and for situational factors in Table 2.

**TABLE 1:** Descriptive statistics for the individual factors.

Measure	M (*n* = 85)	SD	Range
Life satisfaction	24.71	5.52	11–35
Negative emotions in the past 2 weeks	23.22	7.42	10–37
Positive emotions in the past 2 weeks	32.28	7.42	16–49
Peace of mind	3.25	0.72	1.71–5
Anxiety symptoms in the past 2 weeks	5.05	4.12	0–18
Depression symptoms in the past 2 weeks	5.72	4.93	0–22

**TABLE 2:** Descriptive statistics for the situational factors.

Measure	M (*n*)	SD	Range
Negative emotions during mind wandering	2.62 (963)	1.02	1–5
Positive emotions during mind wandering	2.83 (961)	1.02	1–5
Daily negative emotions	16.93 (972)	5.78	9–43
Daily positive emotions	26.48 (972)	8.48	10–50
Daily COVID‐19‐related worry	2.03 (957)	0.92	1–5
Daily COVID‐19‐related media consumption	2.35 (971)	0.90	1–5

### 3.2 Threatening Events

**FIGURE 1:**
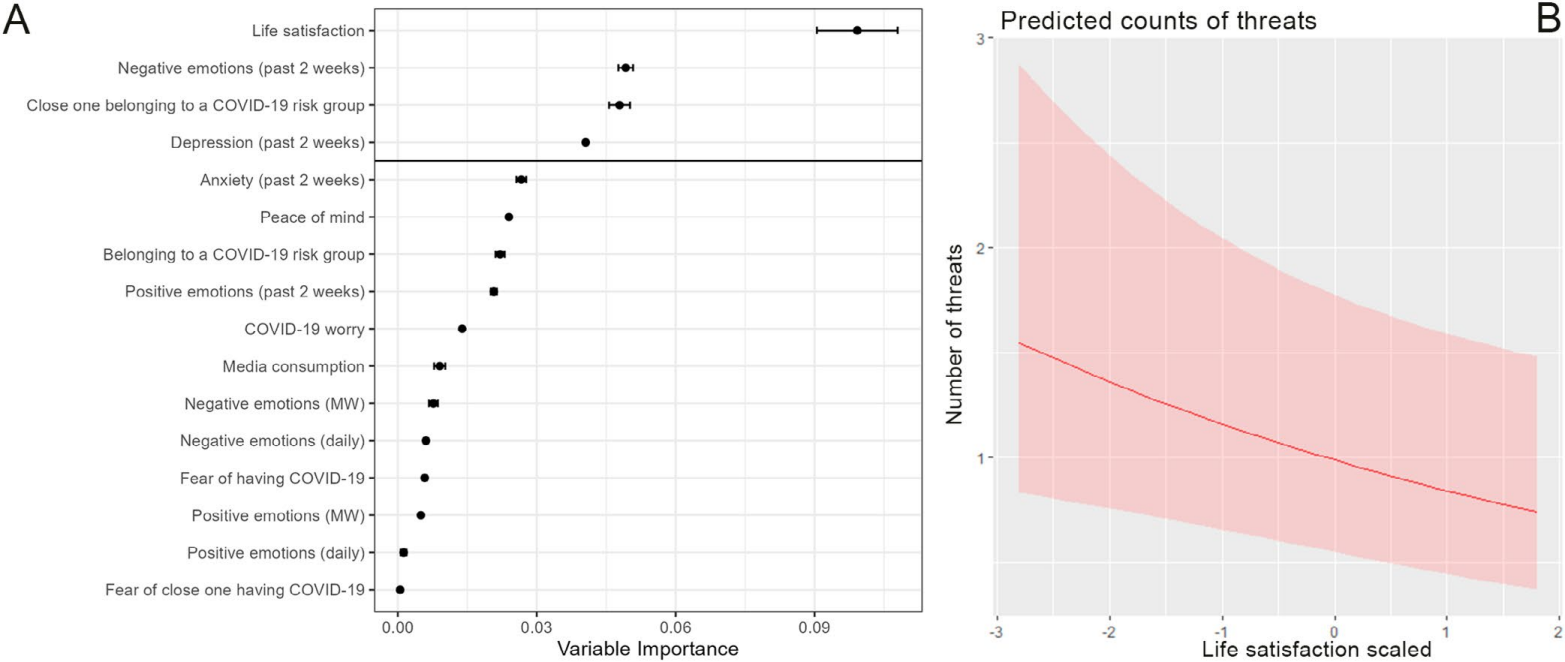
Model results for threatening dream events. (A) Variable importance from the random forest analysis. The horizontal line marks the visually determined threshold for selecting predictor variables for further analysis. (B) Model estimates for the effect of life satisfaction. Values are log‐back‐transformed; shaded areas represent 95% confidence intervals.

Random forest analysis identified the following best predictors of threatening dream events: *life satisfaction*, *negative emotions in the past 2 weeks*, *close one belonging to a COVID‐19 risk group*, and *depression symptoms in the past 2 weeks* (Figure 1A). These variables were fitted in the model on *threatening events in dreams* (VIF < 1.6, indicating no signs of multicollinearity). The model revealed an effect of *life satisfaction*, indicating that participants with lower satisfaction had more threatening events in dreams, *β* = −0.16, 95% CI [−0.30, −0.02], *z* = −2.22 (Figure 1B). The model did not show any other effects.

### 3.3 Pandemic‐Related Threatening Events

**FIGURE 2:**
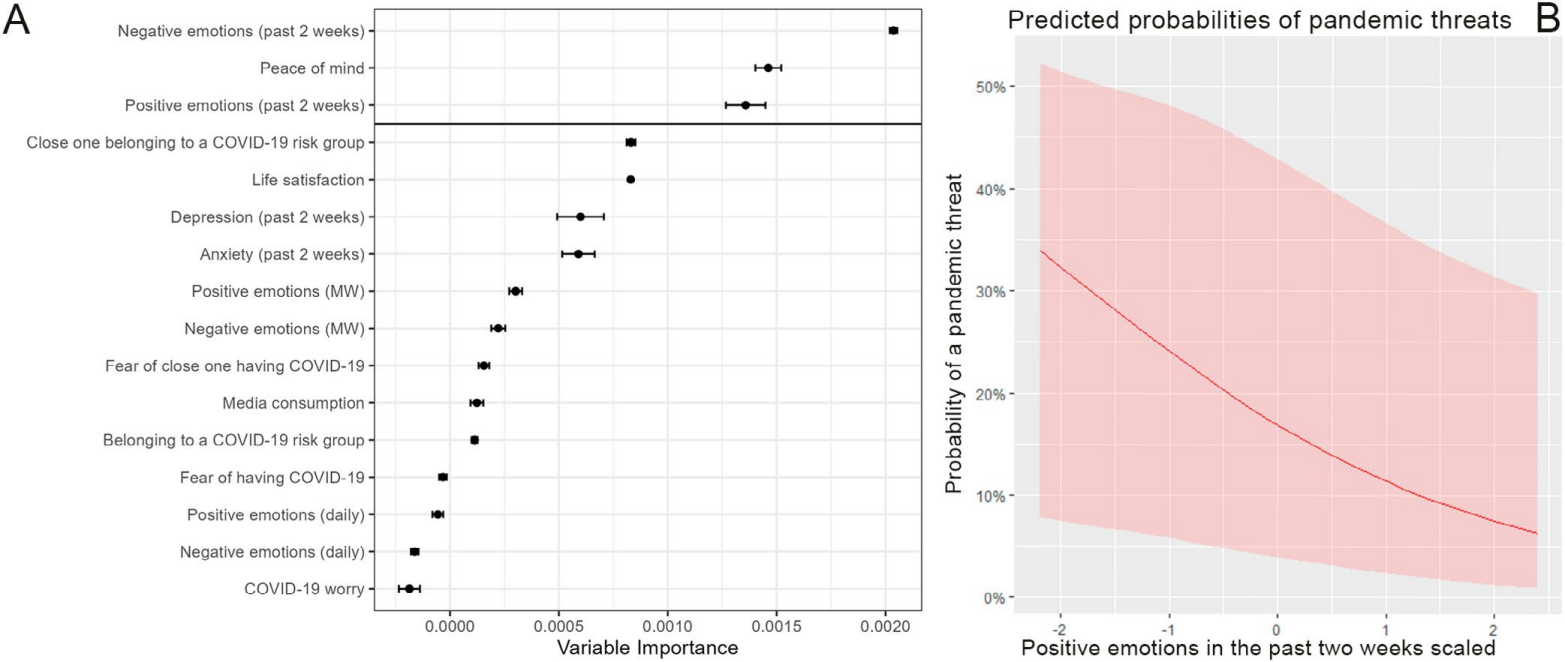
Model results for pandemic‐related threatening dream events. (A) Variable importance from the random forest analysis. The horizontal line marks the visually determined threshold for selecting predictor variables for further analysis. (B) Model estimates for the positive emotions in the past 2 weeks. Values are log‐back‐transformed; shaded areas represent 95% confidence intervals.

Random forest analysis identified the following best predictors of pandemic‐related threatening dream events: *negative emotions in the past 2 weeks*, *peace of mind*, and *positive emotions in the past 2 weeks* (Figure 2A). These variables were fitted in the model on *pandemic‐related threatening events in dreams* (VIF < 1.8, indicating no signs of multicollinearity). The model revealed an effect of *positive emotions in the past 2 weeks*, indicating that participants reporting fewer positive emotions had more pandemic‐related threatening events in dreams, *β* = −0.47, 95% CI [−0.93, −0.01], *z* = −2.00 (Figure 2B). The model did not show any other effects.

## 4 Discussion

We studied which individual and situational factors are the best predictors for threatening events, especially pandemic‐related threatening events, in a large sample of Finnish prospective dream diary dreams. The random forest analyses revealed that almost all the individual factors had higher variable importance for predicting threatening events in dreams and pandemic‐related threatening events in dreams compared to situational factors. This means that the measured individual factors predict threatening dream content better than situational factors. This is in line with previous literature regarding the higher importance of trait features compared to state characteristics as predictors for nightmares and affective dream content (Gratton et al. 2024; Kelly 2025; Samson‐Daoust et al. 2019; Sikka et al. 2023).

However, there is a multitude of research on how waking‐life threatening events predict threatening dream content during the upcoming night (e.g., Bradshaw et al. 2016; Lafrenière et al. 2018; Mellman et al. 2001; Propper et al. 2007). This effect has also been successfully replicated in an experimental setting (Feng and Wang 2022). Notably, higher threat exposure during ontogenetic development seems to alter dream content towards more threat‐related content (Valli et al. 2005). We argue that trait factors are likely to moderate how state factors affect threatening dream content (although there is also opposing evidence, see Schredl 2003). This would explain why, in this study, individual factors had better predictive power than situational factors, but this does not mean that situational factors are unimportant: Rather, situational factors play a role in affecting threatening dream content, but the extent of the effect is dependent on individual factors (similarly as in Blagrove and Fisher 2009).

In this study, lower *life satisfaction* was the only statistically significant predictor of threatening events in dreams. Life satisfaction is considered a stable trait that can be temporarily altered by environmental and personal changes, such as the COVID‐19 pandemic itself and the strict restrictions it might have induced, but after some time, typically returns to the original level (Gnambs and Buntins 2017). Considering that life satisfaction in our sample was very close to average (Pavot et al. 1991), the COVID‐19 pandemic apparently did not have a strong effect on the well‐being of the majority of participants in this study. This does not, however, preclude the possibility that some participants were not affected and might have evaluated their life satisfaction to be higher before the pandemic. A question arises as to why lower life satisfaction is a much better predictor than all the other variables in the random forest analysis and the only significant predictor of threatening events. It has been shown that self‐reported psychological well‐being, similarly to life satisfaction, is a stable trait over at least a 6–to 10‐year period, and lower self‐reported psychological well‐being correlates with more aggressive and emotionally negative dream content over the same time period (Pesant and Zadra 2006). It has also been shown that life satisfaction is associated with sleep quality (Ness and Saksvik‐Lehouillier 2018) and poor sleep quality is associated with more negative and less positive dream affect and nightmares (Sikka et al. 2023). However, in our previous study on the same dream sample collected during the pandemic, sleep quality did not correlate with threatening events (Loukola et al. 2024).

*Positive emotions during the past 2 weeks* were the only statistically significant predictor of pandemic‐related threatening events. Lower positive emotions during the past 2 weeks, evaluated at the beginning of the study, were related to a higher number of pandemic‐related threatening events in the subsequent dreams. This result showcases that a more stable emotional trait is a better predictor of threatening dream content than daily fluctuating emotional states. It is unknown why lower positive emotions, but not higher negative emotions, were a statistically significant predictor of pandemic‐related threatening events. Ke et al. (2022) theorise that positive emotions play a particularly important role when coping with the changes induced by the COVID‐19 pandemic. They found that positive emotions moderate the effect of depression and anxiety on psychological well‐being. In light of our results, it could be speculated that positive emotions protect from the negative psychological effects of the COVID‐19 pandemic, and therefore lower positive emotions predict more pandemic‐related threatening events in dreams.

Both evaluating one's life‐satisfaction and positive emotions in the past 2 weeks require complex memory, metacognitive skills, and self‐awareness compared to evaluating one's current emotional state. Retrospective self‐assessments tap into *beliefs* about oneself rather than the current *experiencing* or even *remembering* self (Kahneman and Riis 2005). If such retrospective evaluation is very negative in nature, this could result in more negative spontaneous thinking. This kind of spontaneous thought can be seen more as a stable trait than a fluctuating state. Such negative daytime thinking could then be reflected in dream content by activating more negative autobiographical memories and therefore more threat simulations. Threatening or otherwise negatively perceived daytime events can add up to the baseline negative thinking, resulting in even more threat simulations. According to TST, individuals develop this baseline through their genetic predispositions and developmental environment, which then enhanced fitness in ancestral environments. This development also makes the threat simulation mechanisms of some individuals more sensitive to detect potential or imagined threat cues in the environment, for example, through trait neuroticism or boundary thinness, which are associated with nightmares (Kelly and Mathe 2024; Schredl and Göritz 2021). Boundary thinness (as opposed to boundary thickness) is a personality trait that is characterised by a tendency to have feelings, narratives, relationships, or concepts to be easily blended with each other (Hartmann 1991).

The main strength of the current study is the large sample size, collected during a relatively short time span that was limited to the harshest COVID‐19 pandemic restrictions in Finland. For example, during this period, schools and public facilities were closed, gatherings were limited to 10 people, people over 70 years old were instructed to avoid all human contact, visitors were not allowed in hospitals or care homes, and citizens and permanent residents returning to Finland were placed under a 2‐week quarantine. Second, we utilised random forest analyses to compare multiple individual and situational factors simultaneously, a methodologically innovative approach not previously applied in dream research. Yet, the study also has some limitations. First, not every dream was accompanied by a unique situational measurement, as situational factors were measured once in the evening after the mind‐wandering task, and some nights could include multiple dream reports. Thus, the results should be replicated before establishing strong conclusions. Second, while TST served as the theoretical background for this study, it makes no explicit predictions about the relative strength of the effects of individual and situational factors on threatening dream content. Additionally, many other theories about the function of dreaming can make the same predictions, such as the continuity hypothesis (Schredl and Hofmann 2003) or emotion regulation theories (Cartwright 2012). The current study will hopefully guide hypothesis formation in future research and theory development. Third, it is unknown how the daily mind‐wandering or dream diary habit might have influenced dream content: keeping a dream diary increases dream recall in poor and moderate recallers compared with retrospective estimates (Schredl 2002), but the effects on dream content are not known. Fourth, by comparing situational factors only to the dream content of the next night, we have not taken into account the possible dream lag effect (Nielsen and Powell 1992). Additionally, dreams not recalled in the morning can be recalled spontaneously during the day (Schredl 2025), but in our instructions to the participants, it was not explicitly mentioned to report these later remembered dreams, even though they had a chance to report their dreams at any time. Fifth, the healthcare personnel who treated COVID‐19 patients should have been included in the COVID‐19 risk group because of their elevated risk of getting COVID‐19, but we had no such inclusion criterion.

In sum, our large dream diary sample, collected during the harshest pandemic restrictions, evidences that, in general, more stable personality trait‐like individual factors are better predictors of threatening events and pandemic‐related threatening events in dreams than situational factors. This indicates that threatening dream content is likely a stable feature of one's psychological make‐up instead of being significantly affected by normally fluctuating daily variables, although it is highly likely that the effect of fluctuating daily variables on threatening dream content is moderated by trait factors. Further research is needed to replicate these findings and investigate how trait factors moderate the effect of state factors on threatening dream content.

## Author Contributions

**Ville Loukola:** conceptualization, writing – original draft, writing – review and editing, data curation, formal analysis, methodology, project administration, resources, investigation. **Jarno Tuominen:** data curation, investigation, writing – review and editing. **Eveliina Malinen:** formal analysis, writing – review and editing. **Katariina Tienhaara:** formal analysis, writing – review and editing. **Henri Olkoniemi:** methodology, supervision, writing – original draft, writing – review and editing. **Antti Revonsuo:** conceptualization, methodology, supervision, writing – review and editing. **Katja Valli:** conceptualization, methodology, project administration, supervision, writing – original draft, writing – review and editing.

## Funding

This work was supported by the Suomen Kulttuurirahasto (00240806) Signe ja Ane Gyllenbergin Säätiö (6425), TOP‐Säätiö (20210206) and the Turku University Foundation (081199) (V.L.).

## Disclosure

The authors have nothing to report.

## Ethics Statement

Because all the data were collected anonymously, no pre‐evaluation was needed according to the Finnish National Board on Research Integrity.

## Conflicts of Interest

The authors declare no conflicts of interest.

## Supporting information


**Table S1:** Correlation matrix for independent variables and final models for each measure.
**Table S2:** Model for threatening events.
**Table S3:** Model for pandemic related threatening events.

## Data Availability

The data that support the findings of this study are openly available in Open Science Framework at https://osf.io/nq2dh/overview?view_only=1dce49b5df9a4c5daeb61b2032f42847
