# Supplementary material for: Individual and Situational Predictors of Threatening Dream Content During the COVID‐19 Pandemic
Source: J Sleep Res. 2026 Apr 2;35(4):e70336. doi: 10.1111/jsr.70336 (PMC13357791; doi:10.1111/jsr.70336)
Supplement: Supplementary file 1 — Table S1: Correlation matrix for independent variables and final models for each measure. Table S2: Model for threatening events. Table S3: Model for pandemic related threatening events. [file JSR-35-e70336-s001.docx]

| **Table S1** | | | | | | | | | | | |
| --- | --- | --- | --- | --- | --- | --- | --- | --- | --- | --- | --- |
|  | Negative emotions during mind-wandering | Positive emotions during mind-wandering | COVID-19 worry | COVID-19 media consumption | Negative emotions today | Positive emotions today | Life satisfaction | Negative emotions in the past two weeks | Positive emotions in the past two weeks | Peace of mind | Anxiety symptoms in the past two weeks |
| Negative emotions during mind-wandering |  |  |  |  |  |  |  |  |  |  |  |
| Positive emotions during mind-wandering | -.43 [-.48, -.38] |  |  |  |  |  |  |  |  |  |  |
| COVID-19 worry | .29 [.23, .35] | -.10 [-0.16, -.04] |  |  |  |  |  |  |  |  |  |
| COVID-19 media consumption | .13 [.07, .19] | .03 [-.03, .09] | .31 [.25, .37] |  |  |  |  |  |  |  |  |
| Negative emotions today | .46 [.41, .51] | -.25 [-.31, -.19] | .37 [.31, .42] | .07 [.01, 0.14] |  |  |  |  |  |  |  |
| Positive emotions today | -.24 [-.30. -.18] | .46 [.41, .51] | .10 [.04, .17] | .01 [-.05, .08] | -.12 [-.18, -.06] |  |  |  |  |  |  |
| Life satisfaction | -.03 [-.09, .03] | .16 [.10, .22] | -.03 [-.09, .03] | .02 [-.04, .08] | -.17 [-.23, -.11] | .22 [.15, .27] |  |  |  |  |  |
| Negative emotions in the past two weeks | .19 [.13, .25] | -.10 [-.16, -.04] | .17 [.10, .22] | .11 [.04, .17] | .43 [.38, .48] | -.17 [-.23, -.10] | -.30 [-.36, -.25] |  |  |  |  |
| Positive emotions in the past two weeks | -.01 [-.07, .05] | .17 [.11, .23] | .11 [.04, .17] | -.05 [-.11, .01] | -.11 [-.17, -.05] | .38 [.33, .44] | .32 [.27, 37] | -.42 [-.47, -.37] |  |  |  |
| Peace of mind | -.09 [-.16, -.03] | .21 [15, .27] | .01 [-.05, .08] | .03 [-.04, .09] | -.26 [-.32, -.20] | .32 [.27, .38] | .60 [.55, .63] | -.56 [-.60, -.51] | .57 [.53, .61] |  |  |
| Anxiety symptoms in the past two weeks | .16 [.10, .22] | -.12 [-.18, -.06] | .15 [.09, .21] | .11 [.05, .17] | .28 [.22, .34] | -.25 [-.31, -.19] | -.26 [-.32, -.21] | .78 [.75, .80] | -.5 [-.54, -.45] | -.51 [-.55, -.47] |  |
| Depression symptoms in the past two weeks | .17 [.11, .23] | -.14 [-.20, -.07] | .12 [.05, .18] | .04 [-.02, .10] | .18 [.12, .24] | -.27 [-.33, -.21] | -.43 [-.47, -.38] | .48 [.44, .53] | -.37 [-.42, -.32] | -.39 [-.44, -.34] | .65 [.62, .68] |

**Supplement**

Correlation matrix for independent variables and final models for each measure

| *Note.* [95% CI]. Variables belonging to a COVID-19 risk group, a close one belonging to a COVID-19 risk group, fear of having COVID-19, and fear of close one having COVID-19 has been excluded from the table due to being categorical variables. The correlation matrix was calculated using the *psych* package in R statistical software (Revelle, 2025). |
| --- |

| **Table S2**  *Model for threatening events* | | | |
| --- | --- | --- | --- |
| Random effects | *n* | *Variance* | *SD* |
| Participant (intercept) | 85 | 0.21 | 0.46 |
| Fixed effects | *Estimate* | *95% CI* | *z* |
| Intercept | -0.23 | -0.54 — 0.07 | -0.16 |
| Life satisfaction | -0.16 | -0.30 — -0.02 | **-2.22** |
| Negative emotions in the past two weeks | -0.01 | -0.16 — 0.14 | -0.16 |
| Close one risk group | -0.15 | -0.45 — 0.14 | -1.03 |
| Depression symptoms in the past two weeks | -0.02 | -0.16 — 0.13 | -0.25 |
| Age | -0.06 | -0.20 — 0.07 | -0.92 |
| Gender (female vs. grand mean) | 0.06 | -1.29 — 1.17 | -0.10 |
| Gender (male vs. grand mean) | -0.15 | -1.06 — 0.77 | -0.32 |
| *Note.* Statistical significance of p < .05 is indicated by the z-value > \|1.96\|. | | | |

| **Table S3**  *Model for pandemic related threatening events* | | | |
| --- | --- | --- | --- |
| Random effects | *n* | *Variance* | *SD* |
| Participant (intercept) | 84 | 0.79 | 0.89 |
| Fixed Effects | *Estimate* | *95% CI* | *z* |
| Intercept | -2.60 | -3.60 — -1.60 | -5.08 |
| Negative emotions in the past two weeks | 0.08 | -0.36 — 0.53 | 0.37 |
| Peace of mind | 0.47 | -0.08 — 1.00 | 1.69 |
| Positive emotions in the past two weeks | -0.47 | -0.93 — -0.01 | **-2.00** |
| Age | 0.06 | -0.32 — 0.45 | 0.31 |
| Gender (female vs. grand mean) | 0.29 | -3.65 — 4.23 | 0.15 |
| Gender (male vs. grand mean) | 0.34 | -2.74 — 3.42 | 0.22 |
| *Note.* Statistical significance of p < .05 is indicated by the z-value > \|1.96\|. | | | |
